# Fungal formation of selenium and tellurium nanoparticles

**DOI:** 10.1007/s00253-019-09995-6

**Published:** 2019-07-20

**Authors:** Xinjin Liang, Magali Aude Marie-Jeanne Perez, Kenneth Chinedu Nwoko, Philipp Egbers, Joerg Feldmann, Laszlo Csetenyi, Geoffrey M. Gadd

**Affiliations:** 1 Geomicrobiology Group, School of Life Sciences, University of Dundee, Dundee, Scotland DD1 5EH UK; 2 Present Address: Sustainable Environment Research Centre, University of South Wales, Upper Glyntaff, Pontypridd, Wales, CF37 4BD UK; 3 Trace Element Speciation Laboratory (TESLA), Department of Chemistry, University of Aberdeen, King’s College, Meston Walk, Aberdeen, Scotland AB24 3UE UK; 4 Concrete Technology Group, Department of Civil Engineering, University of Dundee, Dundee, Scotland DD1 4HN UK

**Keywords:** Selenium, Tellurium, Nanoparticles, Fungi, Bioreduction, Biorecovery

## Abstract

The fungi *Aureobasidium pullulans*, *Mortierella humilis*, *Trichoderma harzianum and Phoma glomerata* were used to investigate the formation of selenium- and tellurium-containing nanoparticles during growth on selenium- and tellurium-containing media. Most organisms were able to grow on both selenium- and tellurium-containing media at concentrations of 1 mM resulting in extensive precipitation of elemental selenium and tellurium on fungal surfaces as observed by the red and black colour changes. Red or black deposits were confirmed as elemental selenium and tellurium, respectively. Selenium oxide and tellurium oxide were also found after growth of *Trichoderma harzianum* with 1 mM selenite and tellurite as well as the formation of elemental selenium and tellurium. The hyphal matrix provided nucleation sites for metalloid deposition with extracellular protein and extracellular polymeric substances localizing the resultant Se or Te nanoparticles. These findings are relevant to remedial treatments for selenium and tellurium and to novel approaches for selenium and tellurium biorecovery.

## Introduction

Selenium and tellurium are metalloids with chemical properties similar to sulfur, also belonging to Group 16 of the Periodic table, and both commonly occurring in − II, + IV and + VI oxidation states (Eswayah et al. 2016). Selenium and tellurium, and their related compounds, have drawn significant attention in recent years due to their properties of photoconductivity and thermoconductivity. They are classified as ‘E-tech’ elements and are essential components of photovoltaic solar panels, alloying agents, semiconductors and various electronic devices being used as, e.g. copper-indium-gallium-(di)selenide (CIGS) and cadmium telluride (CdTe) (Ramanujam and Singh 2017). Se and Te are usually recovered as by-products, mostly from the anodic slimes associated with electrolytic refining of copper. Electrolytic refining employs a sulfate-based electrolyte to plate copper onto a cathode. However, this electrolyte does not dissolve base metals which leads to accumulation of, e.g. gold, tellurium, selenium, bismuth, silver and other metals, along with various refractory components at the bottom of the electrolytic cells (George 2004; Bullock et al. 2017). Remaining substrates from copper mining and refining may contain selenium at various concentrations ranging from 10–40%. Tellurium concentrations are usually much lower, being around 5% maximum (George 2004). Only 15% of the 2700 tonnes of selenium produced each year is recycled, and most waste selenium products are discharged directly and/or indirectly into the environment, which may pose a threat to both aquatic and terrestrial environments and organisms (Haug et al. 2007).

With increasing demand and more stringent discharge regulations to limit the discharge of Se- and Te-containing wastes, the application of microbial bioprocessing could play an important role by offering cost-effective, and less chemically−based approaches. Furthermore, because of their relatively scarce abundance and low concentrations in geological repositories, in contrast to their high demand and potential in new technologies, and current drawbacks in traditional physico-chemical extraction methods, an understanding of microbial biorecovery of selenium and tellurium is of growing importance (Hunter and Manker 2009; Jain et al. 2014; Jacob et al. 2016).

Microorganisms are capable of transforming a range of selenium and tellurium species through oxidation, reduction, methylation and demethylation (Gadd 1993; Jacob et al. 2016; Eswayah et al. 2016). Filamentous fungi are capable of intracellular and extracellular synthesis of selenium nanoparticles, the latter making bioprocessing and biomass handling easier and providing some advantages over bacteria and other unicellular organisms (Mandal et al. 2006). The large amounts of extracellular enzymes and reductive proteins produced by fungi also provide a means for Se and Te bioreduction and biorecovery (Gharieb et al. 1995, 1999; Gharieb and Gadd 2004; Espinosa-Ortiz et al. 2015a,b, 2016a,b,c, 2017). Several naphthoquinone and anthraquinone compounds produced by *T. harzianum* were reported to have good reducing properties (Liu et al. 2007), and this organism was used for biomass-free extracellular synthesis of silver nanoparticles (Ahluwalia et al. 2014). *Pseudomonas* sp. (Hunter and Manter 2009), *Alternaria alternata* (Sarkar et al. 2012), *Phanerochaete chrysosporium* (Espinosa-Ortiz et al. 2015a,b) and *Lentinula edodes* (Vetchinkina et al. 2013) were able to generate selenium nanoparticles from the reduction of either selenate or selenite, while *Fusarium* sp., *Penicillium citrinum* (Gharieb et al. 1999), *Saccharomyces cerevisiae* (Ottosson et al. 2010), and *Rhodotorula mucilaginosa* (Ollivier et al. 2011) produced nanoscale elemental tellurium from tellurite. *Phanerochaete chrysosporium* can also produce mixed Se-Te nanoparticles when grown with selenite/tellurite (Espinosa-Ortiz et al. 2017). Metalloid reduction can be efficient and significant amounts of metalloids can be extensively deposited around biomass. This can be more effective for removal from solution than biomethylation which may take extended time periods and result in only small amounts of removal, even from concentrated solutions, and necessitating a further trapping step to recover volatilized methylated derivatives (Brady et al. 1996; Gharieb et al. 1999; Chasteen and Bentley 2003; Nancharaiah and Lens 2015). We can hypothesise therefore that the reduction of soluble Se or Te oxyanions provides a potential route for biorecovery of these elements. Furthermore, deposited elemental forms can be of nanoscale dimensions which imbue other important physical and chemical properties of potential industrial relevance. However, in contrast to Se bioremediation, lower attention has been paid in the context of Se and Te biorecovery. Although it is known that metalloid reduction is a property found widely in microbes (Gadd 1993), detailed selection or identification of fungal species with high metalloid immobilisation efficiencies have received limited attention, nor the physical and chemical conditions necessary for optimal reduction and removal from solution. A biological treatment provides an alternative direction for biorecovery of Se and Te from solution. The use of microorganisms to convert metalloid oxyanions to less toxic elemental forms not only reduces the toxicity and bioavailability of selenium and tellurium, with well-known applications in bioremediation (Gharieb et al. 1995, 1999; Gharieb and Gadd 2004; Espinosa-Ortiz et al. 2015a, b, 2016a, b, c, 2017), but also is potentially useful for biorecovery and production of selenium and tellurium nanoparticles for technological applications (Eswayah et al. 2016; Liang and Gadd 2017).

The aim of this research was to explore the potential of selected fungal strains as selenium- and/or tellurite-reducing organisms. Specific objectives were to determine the influence of Se or Te oxyanions on fungal growth and morphology, to determine the removal efficiency of Se or Te oxyanions from solution and to characterise the products generated by metalloid-reducing fungi.

## Materials and methods

### Organisms and media

To examine the metalloid reduction ability of selected fungal strains, *Aureobasidium pullulans* (IMI 45533) (Mowll and Gadd 1984), *Mortierella humilis* (Linnemann ex W. Gams. TRTC 50620), *Trichoderma harzianum* (MTCC-3841), and *Phoma glomerata* ([Corda] Wollenw. and Hochapfel) were used for experiments. Some of these organisms were previously shown to have significant abilities in toxic metal and metalloid biotransformations (Grondona et al. 1997; Birla et al. 2009; Freitas et al. 2011; Gade et al. 2013; Ahluwalia et al. 2014; Siddiquee et al. 2014; Nandini et al. 2017). *A. pullulans*, *M. humilis*, *T. harzianum* and *P. glomerata* were routinely maintained on malt extract agar (MEA) (Sigma-Aldrich, St. Louis, MO, USA) for agar plate experiments and AP1 medium (detailed composition listed below) for liquid experiments. Sodium selenate anhydrous (Na_2_SeO_4_) (Sigma-Aldrich, St. Louis, MO, USA), sodium selenite (Na_2_SeO_3_) (Sigma-Aldrich, St. Louis, MO, USA) and sodium tellurite (Na_2_TeO_3_) (Alfa Aesar, Lancashire, UK) were used as media additions to examine metalloid oxyanion reduction.

Liquid cultures were maintained in 250-ml Erlenmeyer conical flasks containing 100 ml nutrient medium on an orbital shaking incubator (Infors Multitron Standard, Rittergasse, Switzerland) at 125 rpm at 25 °C in the dark. *A. pullulans*, *M. humilis*, *T. harzianum* and *P. glomerata* were grown on AP1 agar medium for 4 days at 25 °C prior to experimental subculture. AP1 agar medium composed the AP1 media ingredients listed below with 15 g L^−1^ No.1 agar (Oxford Formulation). The agar medium was adjusted to pH 5 using 1 M HCl before autoclaving. AP1 liquid medium consisted of (L^−1^ Milli-Q water) (Merck Millipore, Billerica, Massachusetts, USA): d-glucose 30 g (Merck, Readington Township, NJ, USA), (NH_4_)_2_SO_4_ 5 g (Sigma-Aldrich), KH_2_PO_4_ 0.5 g (Sigma-Aldrich), MgSO_4_·7H_2_O 0.2 g (VWR, Radnor, PA, USA), CaCl_2_·6H_2_O 0.05 g (VWR, Radnor, PA, USA), NaCl 0.1 g (Sigma-Aldrich), FeCl_3_·6H_2_O 2.5 mg (Sigma-Aldrich), and trace metals: ZnSO_4_·7H_2_O 4 mg (VWR, Radnor, PA, USA), MnSO_4_·4H_2_O 4 mg (VWR, Radnor, PA, USA), CuSO_4_·5H_2_O 0.4 mg (VWR, Radnor, PA, USA). Sodium selenite (Na_2_SeO_3_), sodium selenate (Na_2_SeO_4_) or sodium tellurite (Na_2_TeO_3_) were dissolved separately in Milli-Q water and sterilised by membrane filtration (cellulose nitrate, 0.2-μm pore diameter, Whatman, Maidstone, Kent, UK) and added to sterile AP1 medium (121 °C, 15 min) at room temperature to give a final concentration of 1 mM. Individual AP1 media ingredients were autoclaved separately and combined when cool: the media was adjusted to pH 5 using 1 M HCl. For inoculation, ten 6-mm-diameter inoculum plugs were used, taken from the margins of actively growing colonies using sterile cork borers (autoclaved at 121 °C, 15 min).

### Fungal growth in the presence of Na_2_SeO_3_, Na_2_SeO_4_ or Na_2_TeO_3_

*A. pullulans*, *M. humilis*, *T. harzianum* and *P. glomerata* were inoculated on Se- and Te-containing malt extract agar (MEA). Sodium selenite (Na_2_SeO_3_), sodium selenate (Na_2_SeO_4_) or sodium tellurite (Na_2_TeO_3_) were added to MEA at 50–55 °C from a sterile stock solution (1 mol L^−1^) in Milli-Q water prior to setting. Test fungi were grown on MEA plates in the dark at 25 °C for 5 days. Disks (6-mm diameter) were then cut, using a sterile cork borer, from the margins of the actively growing colonies and inoculated in the centre of triplicate test plates containing the appropriate medium without or with Na_2_SeO_3_, Na_2_SeO_4_, or Na_2_TeO_3_ at 1 or 5 mM. All incubations were at 25 °C in the dark at least in triplicate. Colony diameters were measured daily in two directions to give an average diameter and measurements were discontinued when the colonies had reached the edge of the Petri dish. The ability of filamentous fungi to reduce selenite, selenate and tellurite was assessed visually, the degree of red (Se) or black (Te) colouration being used as an indicator of reduction to elemental forms.

### Growth inhibition in the presence of Na_2_SeO_3_ or Na_2_TeO_3_

*A. pullulans, M. humilis*, *T. harzianum* and *P. glomerata* were inoculated on malt extract agar plates and incubated in the dark at 25 °C for 5 days. Wells (6-mm diameter) were then cut in the agar at the margins of the growing colony using a sterile cork borer. 200 μL 20 mM sodium selenite (Na_2_SeO_3_) or sodium tellurite (Na_2_TeO_3_) was added to four such wells located equidistant between the growing colony and the edge of the Petri dish. Fungi grown on MEA plates without any additions were used as controls. All incubations were at 25 °C in the dark. Colour changes and colony diameters were measured daily for a further 5 days. The ability of the fungi to reduce selenite/tellurite was again assessed visually by the degree of red or black colouration.

### pH change, tolerance indices and selenite and tellurite concentrations after fungal growth in Se- or Te-containing media

To examine fungal selenite and tellurite biotransformations, test fungi were grown in 100 ml AP1 liquid medium without or with 1 mM Na_2_SeO_3_ or Na_2_TeO_3_ in 250-ml conical flasks on an orbital shaking incubator at 125 rpm at 25 °C in the dark. Fungal biomass was aseptically harvested after growth for 10, 20 and 30 days by centrifugation at 4000 rpm (4880*g*) for 30 min and washed twice with autoclaved Milli-Q water. The supernatants were further clarified by filtering through cellulose acetate membrane filters (0.2-μm pore diameter, Whatman, Maidstone, Kent, UK) at appropriate time intervals prior to analysis of selenite and tellurite concentrations and pH. Metal tolerance was evaluated using a tolerance index (TI) calculated as follows: (dry weight of Se/Te-exposed mycelium/dry weight of control mycelium). Fungal biomass was oven-dried at 105 °C to constant weight and then ground to a powder using a pestle and mortar (Milton Brook, Sturminster Newton, Dorset, UK).

Supernatants from fungi grown with 1 mM Na_2_SeO_3_ or Na_2_TeO_3_ for 10, 20 or 30 days were analysed for the concentrations of selenite and tellurite remaining in solution using inductively coupled plasma mass spectrometry (ICP-MS). Total concentration measurements were performed using an inductively coupled plasma mass spectrometer 7900 (Agilent Technology, Tokyo, Japan). A solution containing 1 μg L^−1^ of gallium, yttrium, thallium and cerium was used to optimise lens parameters and ensure the best detection limit. Hydrogen was used as the collision gas with a flow rate of 3.5 mL min^−1^ to prevent any interference which could affect selenium measurements. Selenium and tellurium standards (VWR, Radnor, PA, USA) were used at concentrations of 0, 0.05, 0.1, 1, 10, 50 and 100 μg L^−1^ to perform an external calibration. Ge (10 μg L^−1^) was added inline and used as an internal standard to correct possible fluctuation of the plasma. Samples were diluted to fit in the external calibration range and spiked with 100 μL HNO_3_ (70%, analytical reagent grade, Fisher Scientific, Loughborough, UK) to reach a 1%(*v*/*v*) final concentration of HNO_3_. For quality control, several samples were spiked with selenium and tellurium standards and a recovery close to 100% was obtained. All samples and standards were diluted/prepared using deionised water (18 MΩ cm).

### Examination of Se and Te nanoparticles produced by fungi

Nanoparticle formation in association with fungal biomass grown with 1 mM Na_2_SeO_3_ or Na_2_TeO_3_ was examined using scanning electron microscopy. Fungal pellets grown in the presence of Na_2_SeO_3_ or Na_2_TeO_3_ for 30 days were cut in half using a sterile scalpel (Swann-Morton, Sheffield, UK) and fixed in 2.5%(*v*/*v*) triple-distilled glutaraldehyde in 5 mM 1,4-piperazine N,N′ bis (2-ethane sulphonic acid) (PIPES) buffer, pH 7.2, for at least 24 h at room temperature. The pH of 5 mM PIPES was adjusted using 1 M NaOH using a Corning pH meter 120 (Corning Incorporated, Corning, NY 14831, USA). After fixation, samples were rinsed twice in 5 mM PIPES buffer, pH 7.2 (15 min per rinse) and then dehydrated through a graded ethanol series (50–100%(*v*/*v*), 15 min per step). Samples were then critical point dried using a liquid CO_2_ BAL-TEC CPD 0.30 critical point dryer (BAL-TEC company, Canonsburg, USA) and subsequently mounted on aluminium stubs using carbon adhesive tape and stored in a desiccator at room temperature. Prior to electron microscopy, samples were coated with 10 nm Au/Pd using a Cressington 208HR sputter coater (Ted Pella, Inc., Redding, CA, USA) and examined using a Philips XL30 environmental scanning electron microscope (ESEM) (Philips XL 30 ESEM FEG) operating at an accelerating voltage of 15 kV. NPs in the culture supernatants were harvested by centrifugation at speeds up to 4000 rpm (4880 g), each centrifugation step lasting for 30 min until the particles in the supernatant were completely separated from the biomass. Harvested particles were then washed through a graded ethanol series (50–100%(*v*/*v*), 15 min per step), washed with sterile Milli-Q water 3 times, and subsequently mounted on aluminium stubs using carbon adhesive tape and stored in a desiccator at room temperature. Prior to electron microscopy, samples were coated with 5 nm Au/Pd and examined using a Philips XL30 environmental scanning electron microscope (ESEM) operating at an accelerating voltage of 15 kV as described previously.

Nanoparticles formed on fungal hyphae were examined for elemental composition using energy-dispersive X-ray analysis (EDXA) before Au/Pd coating the samples in order to exclude the Au/Pd peaks which overlap P/Cl peaks. Spectra were acquired using a Phoenix EDXA analysis system embedded within the environmental scanning electron microscope (Philips XL30 ESEM FEG) operating at an accelerating voltage of 20 kV. X-ray powder diffraction (XRPD) was also used to examine the products produced. Diffraction patterns were recorded from 3 to 120° 2-θ using Ni-filtered Cu K-alpha radiation, and scanning from 3–120° 2-θ counting for 300 seconds per step on a Panalytical X-pert Pro diffractometer using a X-celerator position sensitive detector. Mineralogical phases were identified with reference to patterns in the International Centre for Diffraction Data Powder Diffraction File (PDF).

### Size determination and yield of Se and Te NPs

To determine Se and/or Te particle size in the culture supernatants, the ICP-MS 7900 (Agilent Technology, Tokyo, Japan) was used in single particle mode. Supernatants were obtained by filtering through cellulose acetate membrane filters (0.2-μm pore diameter, Whatman, Maidstone, Kent, UK) and stored at 4 °C prior to analysis. In order to remove potential interferences, hydrogen was used in the collision cell at 3.5 mL min^−1^. Ionic standard solutions of the targeted elements were analysed at 0, 0.05, 0.1, 1 and 10 μg L^−1^ to determine the counts per second per μg L^−1^ of each isotope measured and allow data processing. In order to calculate the nebulisation efficiency, gold NP reference material RM8013 (60 nm, NIST, US) was analysed, using the same settings, at a concentration of 50 ng L^−1^. All sample dilutions and standards were prepared with deionised water (18 MΩ cm). One isotope was monitored per sample (^78^Se, ^125^Te or ^197^Au) for one minute. Between each sample, a rinse step of 2 min was performed with 1%(*v*/*v*) HNO_3_. The dwell time was set at 0.1 ms and the data interpreted using MassHunter software (Agilent Technology, Tokyo, Japan).

In order to determine amounts of selenium and tellurium taken up by the fungi, selenium and tellurium were extracted from the biomass using an ammonium citrate buffer (pH 10) prepared from citric acid (Sigma-Aldrich Company Ltd., Gillingham, Dorset, UK) and an ammonia solution (28%, BDH Laboratory Supplies. Poole, Dorset, UK) used to adjust the pH. In order to assess elemental concentration, 1 M sodium sulphite was dissolved in citrate buffer to give the following reaction (Aborode et al. 2015):

$$ {{\mathrm{Se}}^0}_{\left(\mathrm{s}\right)}+{{{\mathrm{SO}}_3}^{2-}}_{\left(\mathrm{aq}\right)}\kern0.5em \leftrightarrows \kern0.5em {{{\mathrm{Se}\mathrm{SO}}_3}^{2-}}_{\left(\mathrm{aq}\right)}\;\mathrm{or}\kern0.5em {{\mathrm{Te}}^0}_{\left(\mathrm{s}\right)}+{{{\mathrm{SO}}_3}^{2-}}_{\left(\mathrm{aq}\right)}\kern0.5em \leftrightarrows \kern0.5em {{{\mathrm{Te}\mathrm{SO}}_3}^{2-}}_{\left(\mathrm{aq}\right)} $$

The samples were extracted with regular shaking and vortexed for 2 min every 30 min. After 3.5 h, samples were centrifuged (3500 rpm (4270*g*), 3 min) and the supernatants were collected and stored at 4 °C prior to analysis. In order to assess the amount of selenium and tellurium not extracted, the residues obtained after centrifugation were digested in 5 mL concentrated nitric acid (70%) and 2 mL hydrogen peroxide (30%). Samples were left for 12 h to pre-digest before being open-digested in a CEM Corporation Mars 5 digestion microwave oven (CEM Corporation, Matthews, NC, USA), using a 3 stage temperature program. Stage 1 consisted of a ramp to 50 °C (1600 W), held for 5 min, then stage 2 ramped to 75 °C (1600 W) and held for 5 min. Finally, stage 3 was ramped to 95 °C (1600 W) and held for 30 min when total dissolution of the samples was observed. All samples were allowed to cool to room temperature before analysis. Total concentration analysis was performed using an inductively coupled plasma mass spectrometer 7900 (Agilent Technology, Tokyo, Japan) as described previously.

### Production of extracellular protein and exopolysaccharide during fungal growth in the presence of selenite or tellurite

To examine the possible influence of extracellular protein and exopolysaccharide on the formation of selenium and tellurium NPs, supernatants harvested after growth of selected fungal strains grown in liquid media containing 1 mM Na_2_SeO_3_ or Na_2_TeO_3_ for 30 days were centrifuged (12,000*g*, 20 min) and the supernatants clarified by filtering through cellulose acetate membrane filters (0.2-μm pore diameter, Whatman, Maidstone, Kent, UK). Extracellular protein was determined using the Bradford protein assay (Bio-Rad Laboratories, Inc. Watford, UK) with bovine serum albumin as the standard. Polysaccharide content was determined by the phenol-sulphuric acid method (Dubois et al. 1956), using glucose as the standard.

### Statistical analysis

All data presented are means of at least three replicates: error bars represent one standard error either side of the mean. SigmaPlot, version 12.5, was used to perform statistical analyses. Any difference in means between treatments was assessed using one-way analysis of variance (ANOVA) to a 0.05 significance level.

## Results

### Growth on selenite-, selenate- and tellurite-containing media

**Fig. 1:**
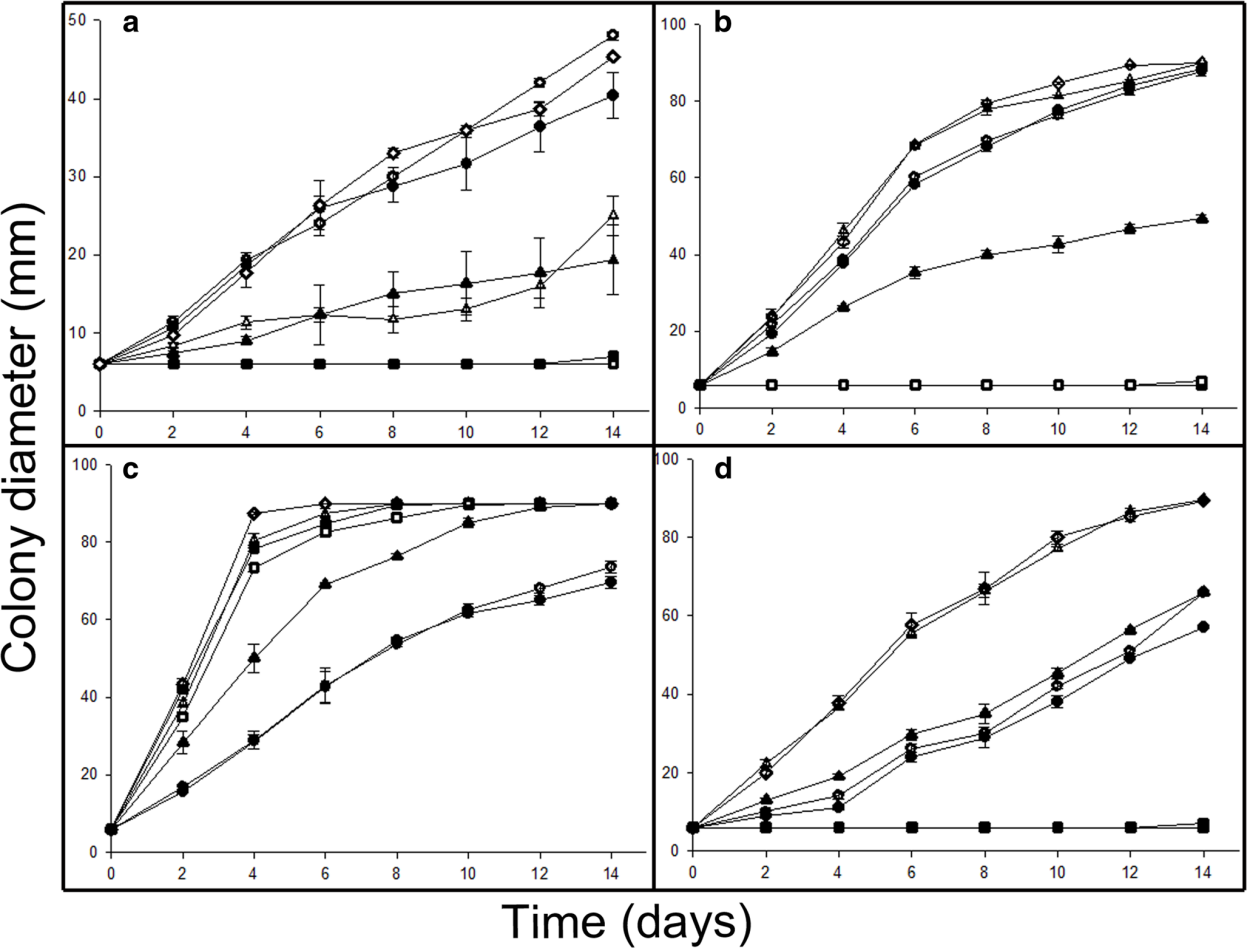
Growth (colony expansion) of test fungi on 1 mM or 5 mM Na_2_SeO_3_-, Na_2_SeO_4_- or Na_2_TeO_3_-amended MEA medium. Fungal colony measurements were carried out every 2 days for (**a**) *A. pullulans* ,(**b**) *M. humilis*, (**c**) *T. harzianum* and (**d**) *P. glomerata* for 14 days. The graphs show growth on () 1 mM Na_2_SeO_3_, () 5 mM Na_2_SeO_3_, () 1 mM Na_2_SeO_4_, () 5 mM Na_2_SeO_4_, () 1 mM Na_2_TeO_3_ and () 5 mM Na_2_TeO_3_-amended MEA medium and () MEA medium only. All test fungi were grown at 25 °C in the dark. Measurements are the mean of two diameter measurements for at least three replicates and error bars indicate the standard error of the mean

Most of the test fungi were able to grow on MEA amended with 1 mM Na_2_TeO_3_ and Na_2_SeO_3_, showing different degrees of black or red colouration respectively. The toxicity of Na_2_TeO_3_ and Na_2_SeO_3_ was more pronounced at a concentration of 5 mM, as indicated by reduced colony expansion rates and degree of black or red colouration. The effect of Na_2_TeO_3_ on *A. pullulans* and *P. glomerata* (Fig. 1a, d) was much stronger than the effect of Na_2_SeO_3_ at both 1 and 5 mM concentrations. For most fungi, little or no growth occurred in the presence of Na_2_SeO_4_, and only *M. humilis* and *T. harzianum* were able to grow on 1 mM Na_2_SeO_4_-amended MEA. There was no significant difference in growth of *M. humilis* (Fig. 1b) and *T. harzianum* (Fig. 1c) in the presence of 1 and 5 mM Na_2_TeO_3_. Both *A. pullulans* (Fig. 1a) and *P. glomerata* (Fig. 1d) were significantly affected by 5 mM Na_2_TeO_3_.

The presence of Na_2_SeO_3_ and Na_2_TeO_3_ resulted in more than 50% growth inhibition for *A. pullulans* (89% and 53.6%) and *M. humilis* (96.8% and 64.5%) (Table 1). Differing effects between Na_2_SeO_3_ and Na_2_TeO_3_ were pronounced for *P. glomerata*, with 82.1% inhibition by selenite but only 28.6% from tellurite after 5 days (Table 1). *T. harzianum* exhibited tolerance and was little affected by the presence of Na_2_SeO_3_ and Na_2_TeO_3_ with 3% and 4% inhibition respectively (Table 1). Significant selenite and tellurite bioreduction was manifested by the red and black colony expansion rings with a marked reduction in colony expansion rates compared with the metalloid-free controls.

**Table 1:** Growth inhibition (GI) of the selected fungi in the presence of Na_2_SeO_3_ and Na_2_TeO_3_. Growth inhibition (GI) was derived from the diameters of fungal colonies, GI = D(control) − D(Na_2_SeO_3_/Na_2_TeO_3_)/D(control) − D(original) × 100%; D represents the fungal colony diameter; D(control) represents the expansion diameter of fungal colonies without any addition; D(Na_2_SeO_3_ / Na_2_TeO_3_) represents the expansion diameter of fungal colonies with the addition of Na_2_SeO_3_ or Na_2_TeO_3_; D(original) represents the original diameter of fungal colonies. For this assay, 200 μL 20 mM sodium selenite (Na_2_SeO_3_) or sodium tellurite (Na_2_TeO_3_) were added to each of four 6-mm-diameter wells located equidistant between the growing colony and the edge of the Petri dish. All test fungi were grown on MEA plates with or without Na_2_SeO_3_ and Na_2_TeO_3_ for 5 days at 25 °C in the dark. All colony diameters used in the calculations were the means of two measurements for at least three replicates

Organism	Growth inhibition (%)	
	Na_2_SeO_3_	Na_2_TeO_3_
*A. pullulans*	89.0 ± 0.15	53.6 ± 0.08
*M. humilis*	96.8 ± 0.05	64.5 ± 0.07
*T. harzianum*	3.0 ± 0.11	4.0 ± 0.12
*P. glomerata*	82.1 ± 0.04	28.6 ± 0.06

### pH changes and tolerance indices of experimental fungi grown in selenite- or tellurite-amended liquid media

All fungi were able to grow in the presence of 1 mM Na_2_SeO_3_ or Na_2_TeO_3_ over a 30-day incubation period at 25 °C. There were no significant differences in medium pH values on addition of Na_2_SeO_3_ or Na_2_TeO_3_ compared with the controls grown in AP1 medium except for *A. pullulans* and *T. harzianum* where the pH remained acidic ranging from pH 2.3 to 2.4 (Table 2). The medium pH for *M. humilis* grown with 1 mM Na_2_TeO_3_ dropped to pH 2.8 compared with the control pH 3.8 (Table 2). The medium pH for *P. glomerata* grown with 1 mM Na_2_TeO_3_ resulted in a rise to pH 7.2, while in the presence of 1 mM Na_2_SeO_3_, the pH dropped to pH 5.6 compared with the control pH of 6.3 (Table 2).

**Table 2:** Medium pH and tolerance index (TI) for *A. pullulans, M. humilis*, *T. harzianum* and *P. glomerata* grown in AP1 medium amended with 1 mM Na_2_SeO_3_ or Na_2_TeO_3_. All values shown are means of at least three measurements.

Organism	pH of media after 30 days			Tolerance index (Rm:Rc)	
	Control	(+)Na_2_SeO_3_	(+)Na_2_TeO_3_	(+)Na_2_SeO_3_	(+)Na_2_TeO_3_
*A. pullulans*	2.32 ± 0.03	2.32 ± 0.01	2.33 ± 0.02	0.62	0.89
*M. humilis*	3.75 ± 0.03	4.41 ± 0.02	2.79 ± 0.03	2.14	2.65
*T. harzianum*	2.40 ± 0.01	2.36 ± 0.03	2.45 ± 0.02	2.27	2.53
*P. glomerata*	6.34 ± 0.01	5.58 ± 0.02	7.18 ± 0.03	0.79	0.96

In the presence of Na_2_SeO_3_ or Na_2_TeO_3_, growth of *A. pullulans* and *P. glomerata* was reduced, while *M. humilis* and *T. harzianum* showed relatively better tolerance in Se/Te-amended AP1 medium (Table 2). Tolerance indices (TI) were used to compare fungal biomass yields grown in AP1 medium with or without 1 mM Na_2_SeO_3_ or Na_2_TeO_3_ (Table 2). A TI value lower than 1 indicates growth inhibition, a TI value larger than 1 suggests growth stimulation. Biomass yields of *A. pullulans* were markedly reduced in the presence of 1 mM Na_2_SeO_3_, with growth inhibition resulting in the presence of 1 mM Na_2_TeO_3_. There was some reduction of biomass yield for *P. glomerata* over the first 20-day incubation in the presence of Na_2_SeO_3_ or Na_2_TeO_3_, but with a longer incubation time, biomass yields increased (Table 2). *M. humilis* and *T. harzianum* showed higher biomass yields in the presence of Na_2_SeO_3_ or Na_2_TeO_3_ (Table 2).

### Removal of selenite and tellurite from liquid media during fungal growth

**Fig. 2:**
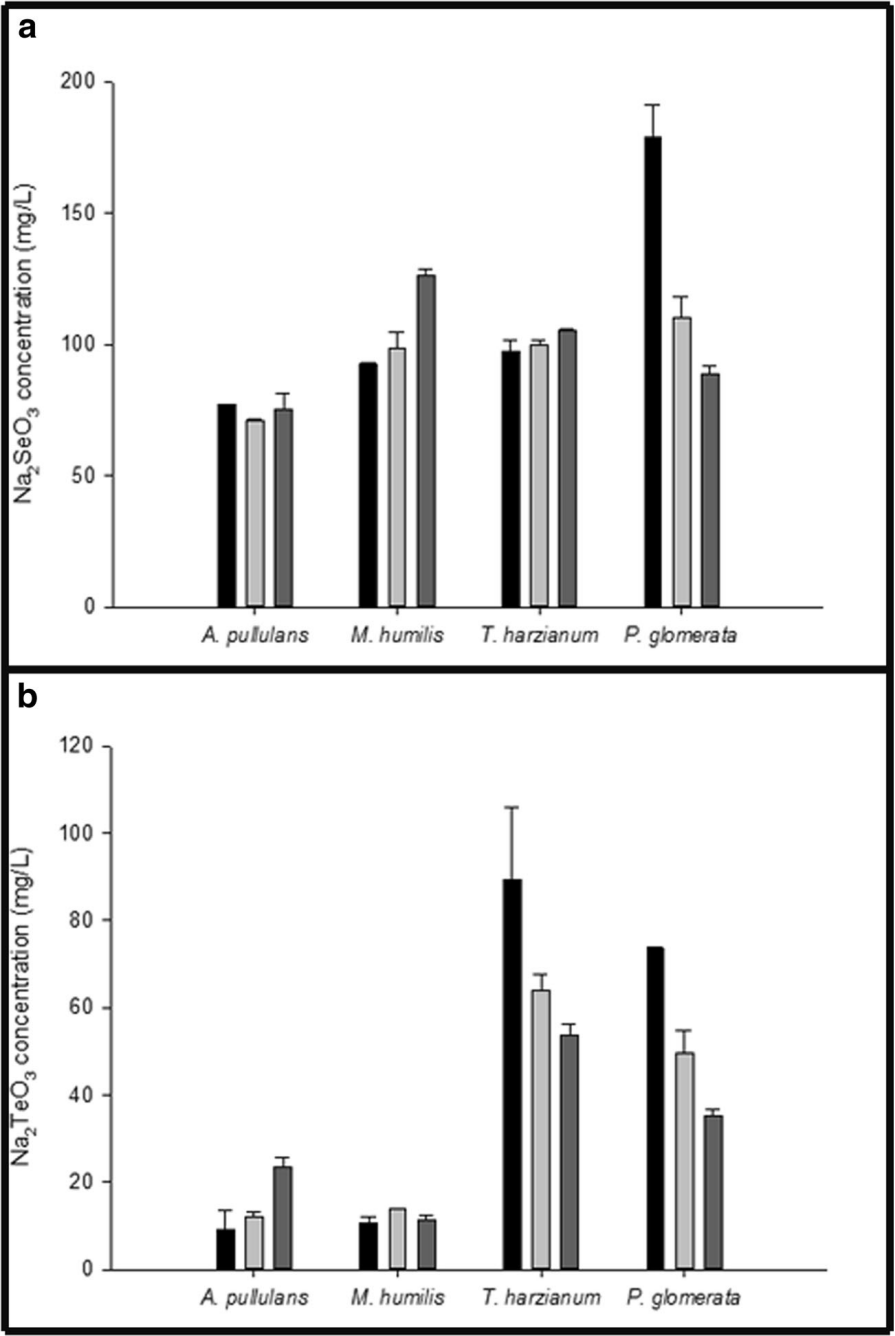
Concentrations of Na_2_SeO_3_ or Na_2_TeO_3_ remaining in culture supernatants after growth of test fungi in 1 mM Na_2_SeO_3_ or Na_2_TeO_3_-amended AP1 liquid medium. (**a**) Na_2_SeO_3_ or (**b**) Na_2_TeO_3_ concentrations remaining in the culture supernatants after growth of *A. pullulans, M. humilis*, *T. harzianum* and *P. glomerata* in AP1 media amended with 1 mM Na_2_SeO_3_ or Na_2_TeO_3_. The bars show Na_2_SeO_3_ or Na_2_TeO_3_ concentrations in the supernatants after growth for () 10 days, () 20 days and () 30 days. All test fungi were grown at 125 rpm 25 °C in the dark. All measurements are from at least three replicates and error bars indicate the standard error of the mean

Fig. 2 shows the concentrations of Na_2_SeO_3_ or Na_2_TeO_3_ remaining in culture supernatants after growth of *A. pullulans*, *M. humilis*, *T. harzianum* and *P. glomerata* in AP1 medium amended with 1 mM (172.9 mg L^−1^) Na_2_SeO_3_ or 1 mM (221.58 mg L^−1^) Na_2_TeO_3_ after 10, 20 and 30 days. There was little difference between the test strains when grown in AP1 medium with 1 mM Na_2_SeO_3_, all fungi being tolerant of this selenite concentration. Remaining concentrations of selenite in the supernatant after growth for 30 days decreased from 1 mM (172.9 mg L^−1^) to around 0.58 mM (100 mg L^−1^) (Fig. 2a). For AP1 medium with 1 mM Na_2_TeO_3_, remaining concentrations of tellurite in the supernatant after growth for 30 days dropped dramatically from 1 mM (221.58 mg L^−1^) to around 0.09 mM (20 mg L^−1^) for *A. pullulans* and *M. humilis,* and 0.23 mM (50 mg L^−1^) for *T. harzianum* (Fig. 2b)*.*

### Formation of elemental selenium and tellurium, and selenium- and tellurium-containing products

**Fig. 3:**
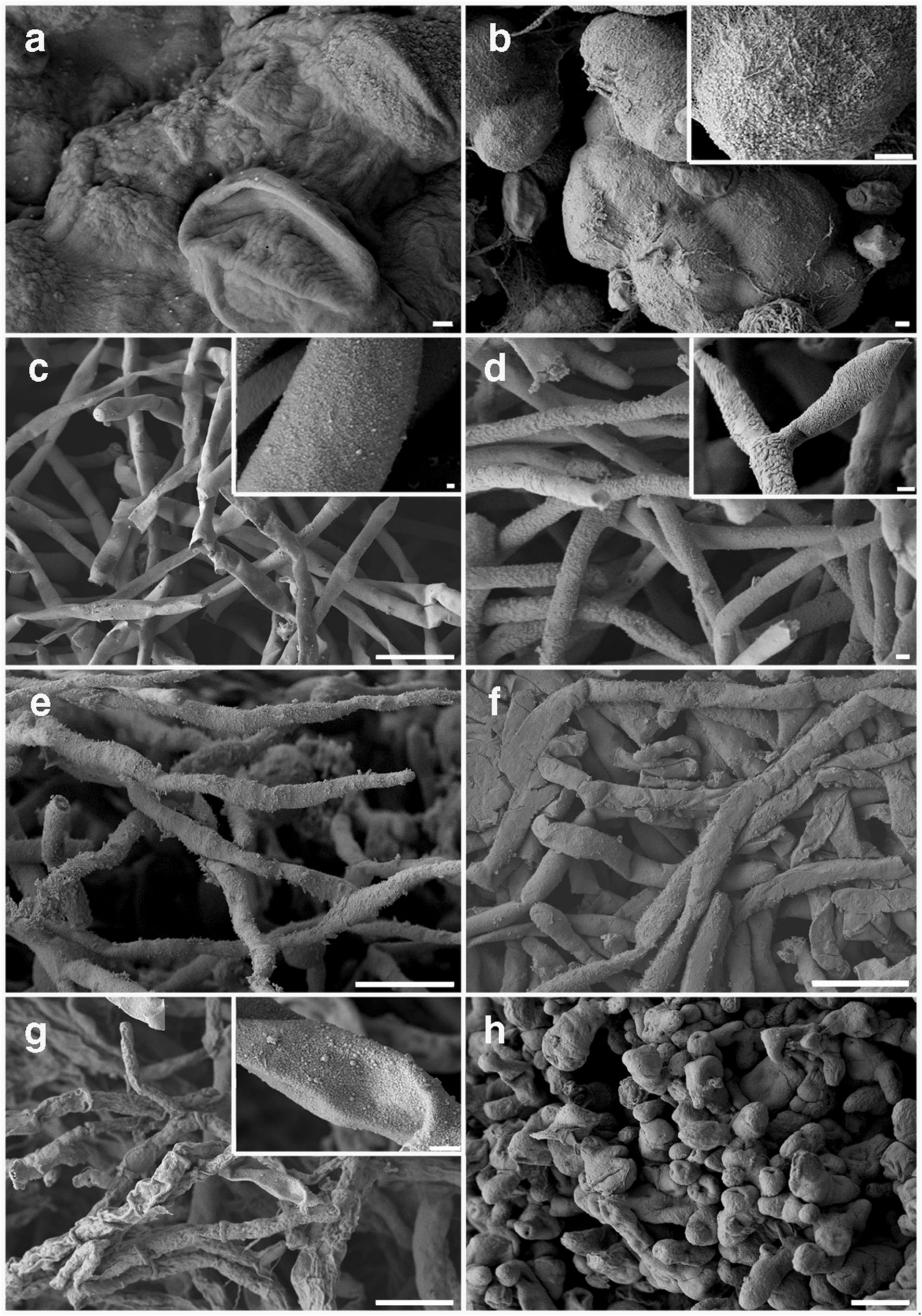
Scanning electron microscopy of nanoparticles formed by *A. pullulans, M. humilis*, *T. harzianum*, and *P. glomerata* grown in AP1 liquid media amended with 1 mM Na_2_SeO_3_ or Na_2_TeO_3_. *A. pullulans* grown in (**a**) 1 mM Na_2_SeO_3_- or (**b**) 1 mM Na_2_TeO_3_-amended AP1 medium. Scale bars: **a**, **b** = 1 μm. The inset in **b** is a higher magnification image of the nanoparticles formed on the fungal surface (scale bar = 1 μm). *M. humilis* grown in (**c**) 1 mM Na_2_SeO_3_-or (**d**) 1 mM Na_2_TeO_3_-amended AP1 medium. Scale bars: **a** = 10 μm, **b** = 1 μm. Insets in **c** and **d** are higher magnification images of the nanoparticles formed on the fungal surfaces (scale bars: c = 100 nm, d = 1 μm). *T. harzianum* grown in (**e**) 1 mM Na_2_SeO_3_- or (**f**) 1 mM Na_2_TeO_3_-amended AP1 medium. Scale bars: **e**, **f** = 10 μm. *P. glomerata* grown in (**g**) 1 mM Na_2_SeO_3_- or (h) 1 mM Na_2_TeO_3_-amended AP1 medium. Scale bars: **g**, **h** = 10 μm. The inset in **g** is a higher magnification image of the nanoparticles formed on the fungal surface (scale bar = 1 μm). All organisms were grown for 30 days at 25 °C in the dark on an orbital shaking incubator at 125 rpm. Typical images are shown from one of at least three examinations

**Fig. 4:**
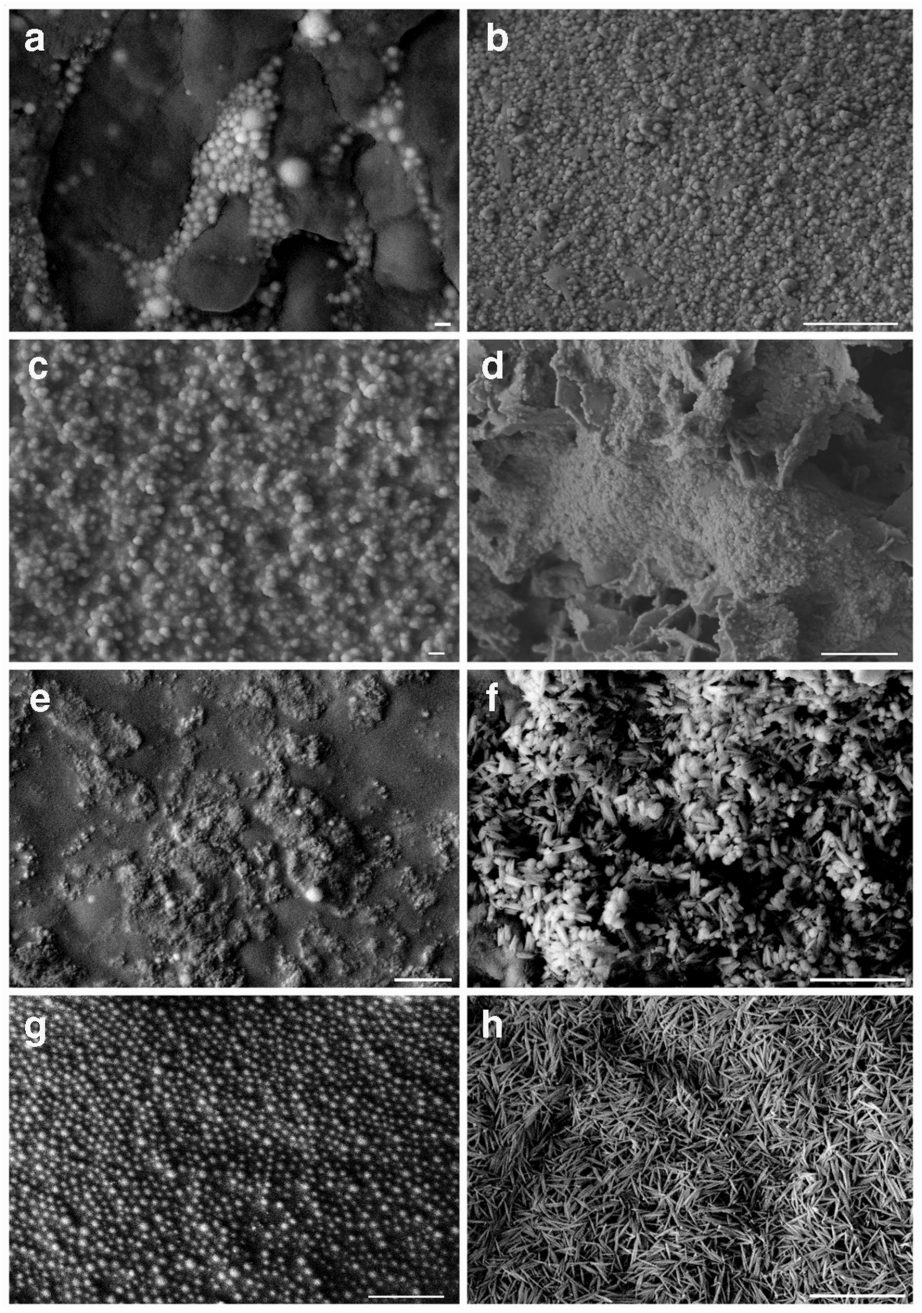
Scanning electron microscopy of nanoparticles harvested from supernatants of *A. pullulans*, *M. humilis*, *T. harzianum* and *P. glomerata* after growth with 1 mM Na_2_SeO_3_ or 1 mM Na_2_TeO_3_. Nanoparticles harvested from the supernatant of *A. pullulans* grown in (**a**) 1 mM Na_2_SeO_3_- or (**b**) 1 mM Na_2_TeO_3_-amended AP1 medium. Scale bars: **a** = 100 nm, **b** = 1 μm. Nanoparticles harvested from the supernatant of *M. humilis* grown in (**c**) 1 mM Na_2_SeO_3_- or (**d**) 1 mM Na_2_TeO_3_-amended AP1 medium. Scale bars: **c** = 100 nm, **d** = 1 μm. Nanoparticles harvested from supernatant of *T. harzianum* grown in (**e**) 1 mM Na_2_SeO_3_- or (**f**) 1 mM Na_2_TeO_3_-amended AP1 medium. Scale bars: **e**, **f** = 1 μm. Nanoparticles harvested from the supernatant of *P. glomerata* grown in (**g**) 1 mM Na_2_SeO_3_- or (**h**) 1 mM Na_2_TeO_3_-amended AP1 medium. Scale bars: **g**, **h** = 1 μm. Typical images are shown from one of at least three examinations

Nanosized particles formed on fungal surfaces and in the medium after growth of *A. pullulans*, *M. humilis*, *T. harzianum*, and *P. glomerata* with 1 mM Na_2_SeO_3_ or Na_2_TeO_3_ (Figs. 3 and 4). Nanoparticles generated from *A. pullulans, M. humilis* and *P. glomerata* with Na_2_SeO_3_ were granular with similar sizes (Figs. 3 and 4a, c, g), while particles generated by *T. harzianum* were of variable shapes (Figs. 3 and 4e), most being aggregated on the fungal surfaces (Fig. 3). Particles generated by *A. pullulans, M. humilis*, *T. harzianum* and *P. glomerata* after growth with Na_2_TeO_3_ were variable in shape and size (Figs. 3 and 4b, d, f, h). Particles generated on surfaces and in culture supernatants of *A. pullulans* and *M. humilis* were granular (Figs. 3 and 4b, d); particles harvested from *M. humilis* supernatant were smaller but formed aggregates (Fig. 4d); particles from *A. pullulans* supernatants were of similar size and well dispersed (Fig. 4b). Particles generated from *T. harzianum* and *P. glomerata* comprised pillar and needle shapes of various sizes (Fig. 4f, h), while nanorods harvested from *T. harzianum* supernatants clustered together being composed of numerous individual shards (Fig. 4f).

### Energy-dispersive X-ray analysis

**Fig. 5:**
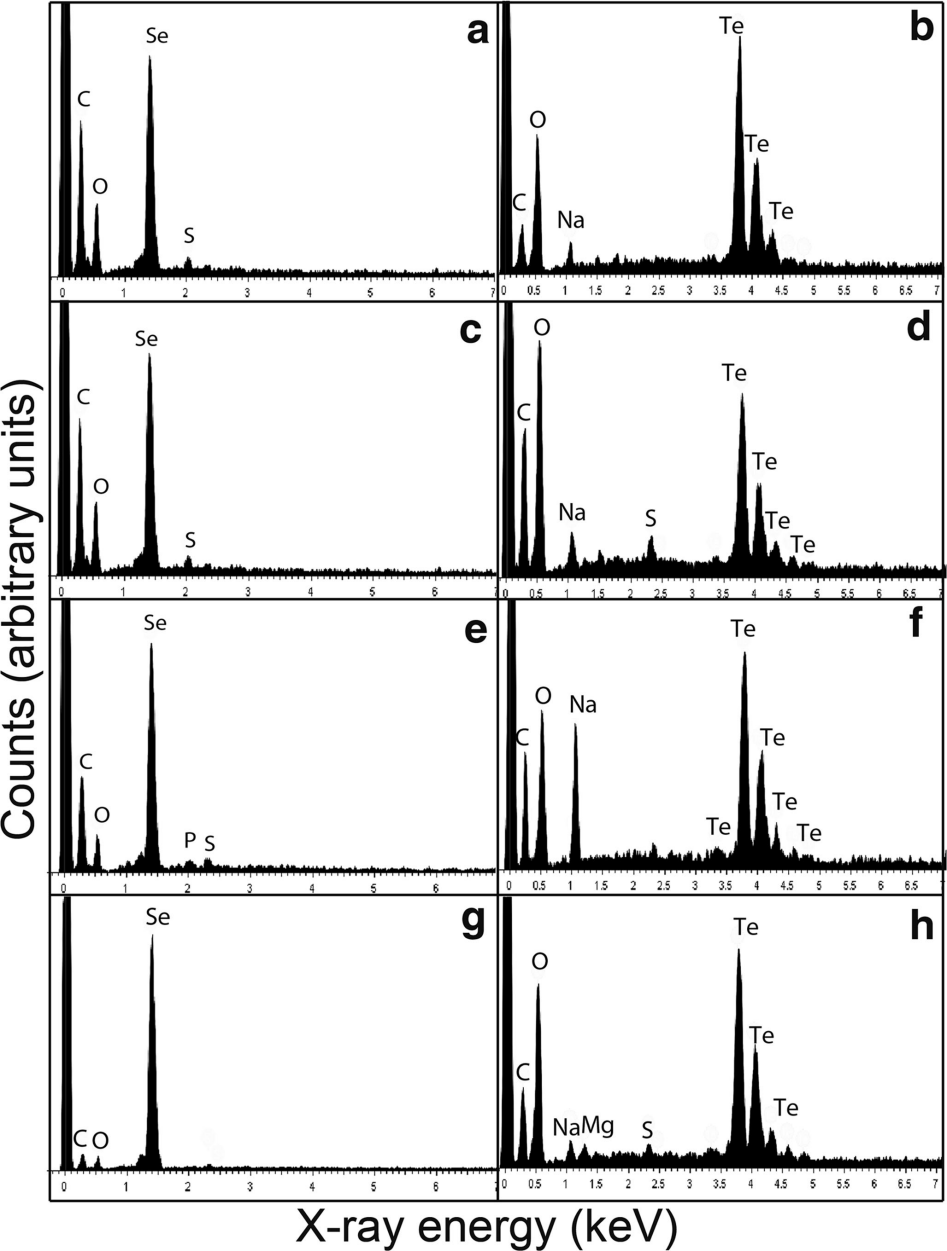
EDXA of nanoparticles produced by *A. pullulans, M. humilis*, *T. harzianum* and *P. glomerata* grown in AP1 liquid media amended with 1 mM Na_2_SeO_3_ or Na_2_TeO_3_ for 30 days at 25 °C in the dark on an orbital shaking incubator at 125 rpm. **a**, **c**, **e**, **g** Selenium-containing particles produced by *A. pullulans, M. humilis*, *T. harzianum* and *P. glomerata* (shown in Fig. 3a, c, e, g). **b**, **d**, **f**, **h** Tellurium-containing particles produced by *A. pullulans*, *M. humilis*, *T. harzianum* and *P. glomerata* (shown in Fig. 3 b, d, f, h). Typical spectra are shown from one of at least three determinations

Energy-dispersive X-ray analysis (EDXA) was used to reveal the elemental composition of the particles produced by the fungi. Most particles generated after fungal growth with 1 mM Na_2_SeO_3_ showed peaks for carbon, oxygen, sulphur and selenium as the main elements (Fig. 5a, c, e, g). Particles generated after growth in AP1 medium amended with 1 mM Na_2_TeO_3_ showed peaks for carbon, oxygen, sodium, sulphur and tellurium as the main elements (Fig. 5b, d, f, h).

### Particle sizes and yields of Se and Te nanoparticles

**Fig. 6:**
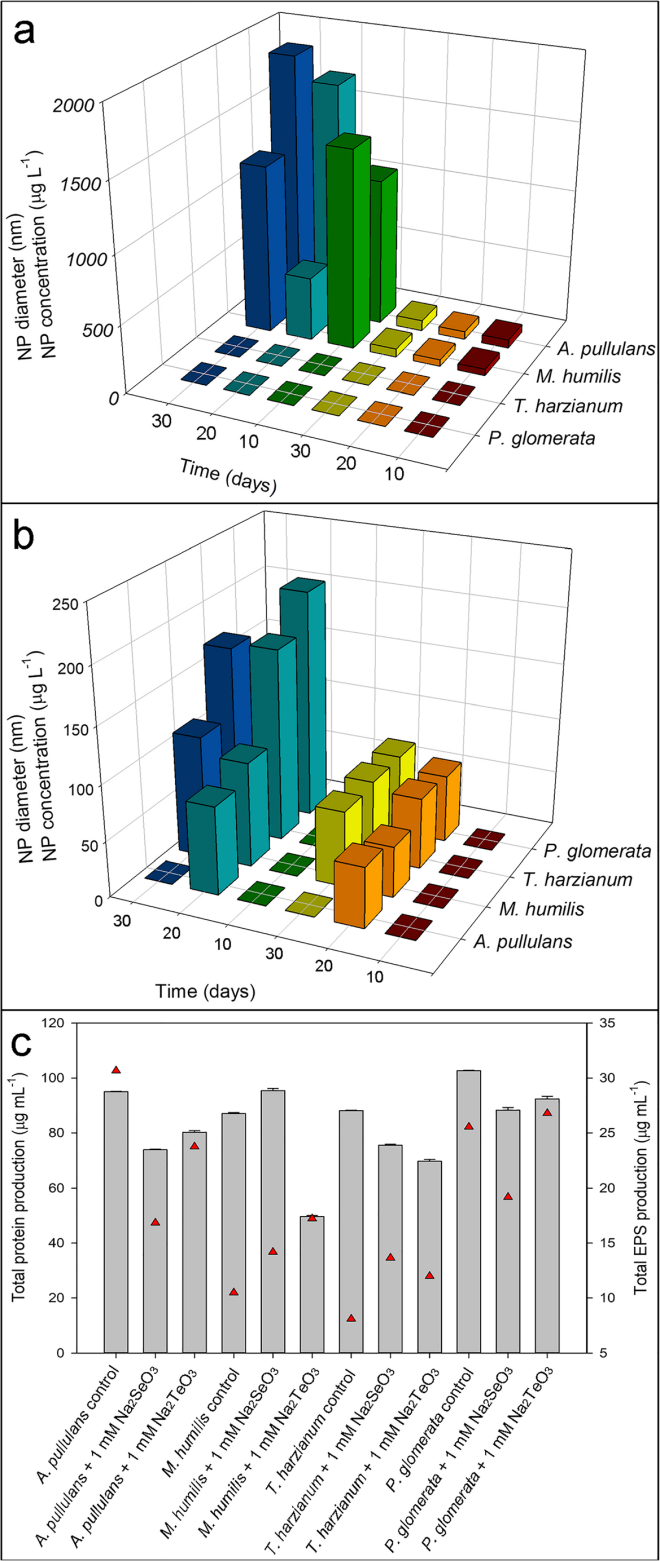
Diameters and concentrations of Se and Te NPs, total extracellular protein and exopolysaccharide production by the test fungi grown in AP1 liquid medium amended with 1 mM Na_2_SeO_3_ or Na_2_TeO_3_. Diameters and concentrations of (**a**) Se and (**b**) Te nanoparticles generated after growth of *A. pullulans, M. humilis*, *T. harzianum*, and *P. glomerata* in the presence of 1 mM Na_2_SeO_3_ or Na_2_TeO_3_. The graphs show diameters of Se and Te NPs after growth for () 10 days, () 20 days and () 30 days, and concentration of Se and Te NPs after growth for () 10 days, () 20 days and () 30 days. **c** Total extracellular protein () and exopolysaccharide () concentration after growth of test fungi with 1 mM Na_2_SeO_3_ or Na_2_TeO_3_. All test fungi were grown in AP1 liquid medium at 125 rpm at 25 °C in the dark. All measurements are from at least three replicates and error bars indicate the standard error of the mean

Both particle diameters and Se and Te NPs concentrations were determined by single particle ICP-MS (Fig. 6). The amounts of Se and Te taken up by the biomass were also determined (Table 3). Only *A. pullulans* and *M. humilis* produced Se nanoparticles after a 10-day incubation, with diameters of ~ 60 nm and ~ 48 nm, and at concentrations of 1079 μg L^−1^ and 1463 μg L^−1^ respectively. After 20 and 30 days incubation, diameters increased to ~ 78 nm and ~ 61 nm, with concentrations of 1885 μg L^−1^ and 1237 μg L^−1^ respectively (Fig. 6a). Low particle concentrations were detected in supernatants of *T. harzianum*, and *P. glomerata* cultures using the single particle analysis method. This may be related to the filtration step before analysis, particles possibly aggregating and being removed by the filtration. Another possibility is that elemental selenium and tellurium associated with biomass surfaces in early growth stages become dissociated after a longer incubation period.

**Table 3:** Se and Te NPs concentration and yield from the test fungi grown with 1 mM Na_2_SeO_3_ or 1 mM Na_2_TeO_3_. All test fungi were grown for 30 days in AP1 media at 125 rpm at 25 °C in the dark. Se/Te extraction concentration indicates Se/Te NP concentration generated by fungi; Se/Te digestion concentration indicates Se/Te taken up by the fungal biomass (as g fresh wt^−1^). All measurements are from at least three replicates

	Se extraction concentration (μg g^−1^)	Se digestion concentration (μg g^−1^)	Se NP yield (%)	Te extraction concentration (μg g^−1^)	Te digestion concentration (μg g^−1^)	Te NP yield (%)
*A. pullulans*	12.0 ± 0.08	38.8 ± 0.11	23.1	20 ± 0.02	10 ± 0.12	66.7
*M. humilis*	55.4 ± 0.65	55.6 ± 0.14	49.9	1500 ± 1.3	790 ± 3.6	65.5
*T. harzianum*	28.8 ± 0.14	40.9 ± 0.37	41.4	1370 ± 3.2	340 ± 4.2	80.1
*P. glomerata*	2.9 ± 0.12	29.6 ± 0.09	8.9	670 ± 2.1	10 ± 0.12	98.5

For particles formed with Na_2_TeO_3_, large differences from those obtained with Na_2_SeO_3_ were observed. Low concentrations of nanoparticles were detected in 10-day-old fungal supernatants grown with 1 mM Na_2_TeO_3_. Nanoparticles occurred in supernatants after a 20-day incubation, with most particle diameters in the range 40–70 nm, with concentrations of particles between 80 and 200 μg L^−1^. *P. glomerata* produced the most Te NPs at more than 200 μg L^−1^, while *A. pullulans* produced the least Te NPs at 79 μg L^−1^. Te NPs concentrations from supernatants of *M. humilis* and *T. harzianum* were 95 μg L^−1^ and 174 μg L^−1^ respectively. After a 30-day incubation, particle diameters and concentrations were similar to those found after 20 days (Fig. 6b).

Amounts of Se and Te associated with fungal biomass were determined after acid digestion and extraction. Se accumulation values for *A. pullulans, M. humilis*, *T. harzianum* and *P. glomerata* were 12, 55, 29 and 3 μg g^−1^, respectively, corresponding to recoveries of 23.1, 49.9, 41.4 and 8.9% (Table 3). Te concentrations were 20, 1500, 1370 and 670 μg g^−1^, respectively, with corresponding recoveries of 66.7, 65.5, 80.1 and 98.5% (Table 3).

### Extracellular protein and exopolysaccharide concentrations

The presence of Na_2_SeO_3_ and Na_2_TeO_3_ had an effect on extracellular protein secretion. Compared with the control, the amount of extracellular protein decreased slightly after growth with 1 mM Na_2_SeO_3_ and Na_2_TeO_3_. Most of the test fungal strains produced similar amounts of protein in control medium at around 90–100 μg mL^−1^ (Fig. 6c). In the presence of 1 mM Na_2_SeO_3_ and Na_2_TeO_3_, the protein concentration for *A. pullulans* dropped to 73 μg mL^−1^ and 80 μg mL^−1^, respectively, from 95 μg mL^−1^; the protein concentration for *T. harzianum* dropped to 75 μg mL^−1^ and 69 μg mL^−1^, respectively, from 88 μg mL^−1^; the protein concentration for *P. glomerata* dropped to 88 μg mL^−1^ and 92 μg mL^−1^, respectively, from 102 μg mL^−1^ (Fig. 6c). There was a significant protein concentration decrease for *M. humilis* from 87 to 49 μg mL^−1^ in the presence of 1 mM Na_2_TeO_3_ (Fig. 6c).

The exopolysaccharide concentration patterns did not correlate with extracellular protein concentration patterns, and varied between different species. *A. pullulans* produced the most exopolysaccharide at 30 μg mL^−1^, while in the presence of 1 mM Na_2_SeO_3_ and 1 mM Na_2_TeO_3_, it dropped to 16 μg mL^−1^ and 23 μg mL^−1^, respectively, after a 30-day incubation (Fig. 6c). Exopolysaccharide production by *M. humilis* was enhanced by the presence of 1 mM Na_2_TeO_3_ yielding 17 μg mL^−1^ (Fig. 6c). Exopolysaccharide production by *T. harzianum* was the lowest compared with the other strains. The concentration of exopolysaccharide from *P. glomerata* with 1 mM Na_2_SeO_3_ and 1 mM Na_2_TeO_3_ were similar to the control (25 μg mL^−1^), being 19 μg mL^−1^ and 26 μg mL^−1^, respectively (Fig. 6c).

### X-ray powder diffraction

**Fig. 7:**
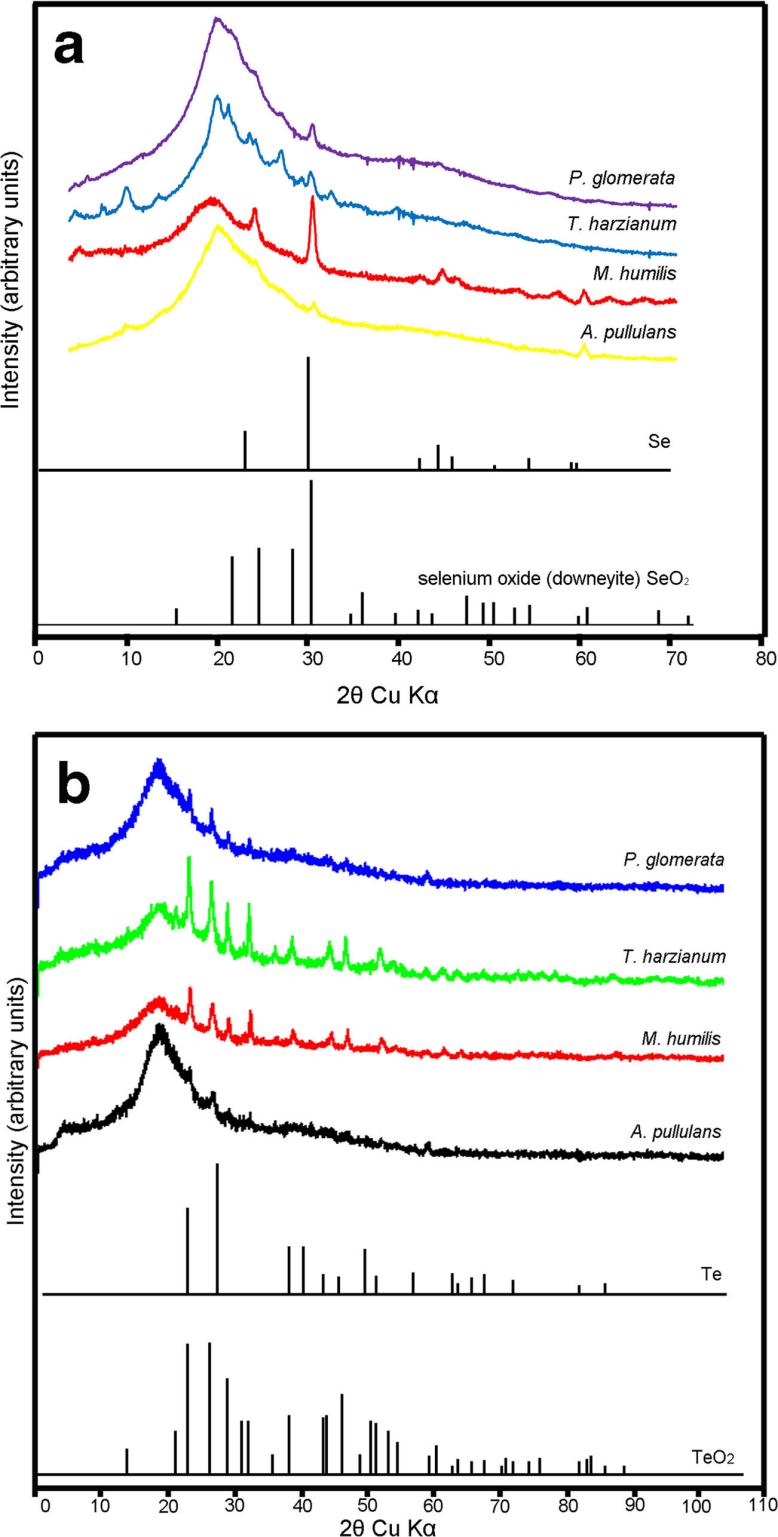
X-ray powder diffraction patterns of particles formed with (**a**) 1 mM sodium selenite- or (**b**) sodium tellurite-amended AP1 liquid medium after fungal growth at 25 °C in the dark at 125 rpm for 30 days. Patterns for dominant components are shown, as well as the particles produced as a result of fungal activity: elemental selenium (Se) and (**a**) downeyite (SeO_2_), elemental tellurium (Te) and (**b**) tellurium oxide (TeO_2_). **a** Diffraction patterns collected from particles harvested from *A. pullulans, M. humilis*, *T. harzianum*, and *P. glomerata* grown with 1 mM Na_2_SeO_3_. **b** Diffraction patterns collected from particles harvested from *A. pullulans*, *M. humilis*, *T. harzianum* and *P. glomerata* grown with 1 mM Na_2_TeO_3_. Typical diffraction patterns are shown from one of several determinations

The selenium-containing particles associated with *A. pullulans*, *M. humilis*, *T. harzianum* and *P. glomerata* showed an excellent match to reference patterns for elemental selenium (Se), while downeyite (SeO_2_) was only detected with *T. harzianum* (Fig. 7a). The tellurium-containing particles associated with *A. pullulans*, *M. humilis*, *T. harzianum* and *P. glomerata* showed elemental tellurium (Te), and tellurium oxide was detected in the particles generated by *T. harzianum* and *M. humilis* (Fig. 7b)*.* The XRD patterns displayed here are consistent with earlier reports (Zare et al. 2012). Other unidentified peaks are possibly due to organic impurities present in the samples, and may indicate the presence of capping agents. Such unidentified peaks in XRD patterns are also apparent in other Te NPs studies (Zare et al. 2012).

## Discussion

### SeO_3_^2−^- and TeO_3_^2−^-reducing microorganisms

This work has shown that *A. pullulans, M. humilis*, *T. harzianum* and *P. glomerata* were capable of reducing both selenite and tellurite to elemental selenium and elemental tellurium respectively. However, not all fungi could reduce selenate (Fig. 1). Biogenic elemental selenium nanoparticles are red in colour while commercial elemental selenium particles of various sizes are black, the colour difference between different selenium particle sizes being attributed to the collective oscillation of free conduction electrons induced by an interacting electromagnetic field, which is called the surface plasmon resonance peak: this phenomenon results from the promotion or excitation of relatively loosely held electrons to higher electrical conductivity (Dwivedi et al. 2011, 2013). The reduction of SeO_3_^2−^ and TeO_3_^2−^ to Se^0^ and Te^0^ was generally accompanied by inhibition of fungal growth as measured by colony expansion (Gharieb et al. 1995, 1999), Among the test fungi, *T. harzianum* apparently showed better selenite and tellurite tolerance according to growth inhibition measurement (Table 1). However, tolerance indices based on biomass yield showed that *M. humilis* and *T. harzianum* produced higher biomass yields when grown with SeO_3_^2−^ and TeO_3_^2−^ comparable to that of the control (Table 2). Previous work showed that *Penicillium citrinum* exhibited no significant decrease in biomass yield in the presence of NaTeO_3_ (127 mg Te L^−1^), although a *Fusarium* sp. did show a significant reduction (Gharieb et al. 1999). Thus, different degrees of inhibition of fungal growth in the presence of SeO_3_^2−^ and TeO_3_^2−^ were detected from both growth inhibition measurement and tolerance indices among the test strains. Two main hypotheses have been proposed for selenium and tellurium biotransformations by microorganisms. Several sulfate-reducing bacteria (e.g. *Desulfovibrio desulfuricans*, *Desulfomicrobium norvegicum*, *Chromatium* spp.) can reduce selenium oxyanions to elemental selenium or produce methylated selenium products during the reduction of SO_4_^2−^ to S^2−^. The reduction rates of SO_4_^2−^ and SeO_4_^2−^ were closely related to their concentrations (Zehr and Oremland 1987; Hockin and Gadd 2003; Baesman et al. 2007). Another work has demonstrated that reduction of selenium oxyanions can occur by pathways separate from sulfate reduction (Oremland et al. 1989, 1994, 2004). Dissimilatory Se-reducing bacteria employ various electron donors to reduce selenium oxyanions, which include sugars, organic acids, alcohols, hydrogen and humic substances (Kashiwa et al. 2000; Zhang et al. 2004, 2008, 2015; Chung et al. 2006; Astratinei et al. 2006).

Previous research has demonstrated that SeO_3_^2−^-, SeO_4_^2−^- and/or TeO_3_^2−^- and TeO_4_^2−^-reducing bacteria are frequently isolated from natural microbial communities (Zhang et al. 2004; Jain et al. 2014, 2015; Tan et al. 2016). The application of microbial consortia for selenium oxyanion removal from contaminated matrices has been developed, such as the ABMet®biofilter system, electro-biochemical reactors (EBR), biofilm reactors (BSeR), membrane biofilm reactors (MBfR), upflow anaerobic sludge blanket reactors (UASB) and sequencing batch reactors (SBR) (Tan et al. 2016). However, potential applications for tellurium oxyanion removal or recovery has received limited attention (Tan et al. 2016). Extracellular polymeric substances including polysaccharides can also play a role in the formation of biogenic elemental selenium and tellurium nanoparticles (BioSeNPs/BioTeNPs). Functional groups characteristic of protein and carbohydrate are found on BioSeNPs, suggesting that a coating formed by EPS can determine colloidal properties and surface charge (Zhang et al. 2004; Jain et al. 2014, 2015). Previous research has also confirmed the complete bioconversion of TeO_3_^2−^ to its elemental state in the form of Te-nanostructures associated with the loosely bound EPS fraction surrounding activated sludge, which suggested a pivotal role played by EPS and its functional groups in the genesis of tellurium nanoparticles (Mal et al. 2017). The appearance of a bright red and black colour indicates the formation of amorphous elemental selenium and tellurium particles. It is likely that selenite and tellurite were reduced through a series of steps. Some of the added selenite or tellurite in the fungal culture medium is taken up by the selected fungi and reduced intracellularly. Reduction to elemental selenium and tellurium also can occur through secreted protein, surface constituents such as glycoproteins, extracellular polymeric substances, such as polysaccharide, protein, nucleic acids, humic substances, with functional groups such as carboxylic, phosphoryl amino and hydroxyl groups (Tsuneda et al. 2003, Guibaud et al. 2008, Holmes and Gu 2016). Extracellular polymeric substances can act as capping agents and control the size and shape of elemental selenium and tellurium NPs, as well as enhancing stability. The stability of Se and Te NPs is important in considering possible toxicity from released ions. In our work, the Se and Te NPs appeared to be stable in the longer term and still retained their shapes and sizes after 6 months. The possibility to remove and recover selenium and tellurium nanoparticles associated with the EPS fraction suggests a new approach for biorecovery of Se or Te.

### Formation of elemental Se, Te, selenium oxide and tellurium oxide

This work has demonstrated the formation of elemental Se and Te by *A. pullulans, M. humilis*, *T. harzianum* and *P. glomerata*, together with selenium- and tellurium-containing oxide nanoparticles in *T. harzianum*, and tellurium-containing oxide nanoparticles in *M. humilis*. Several researchers have investigated mechanisms of microbial formation of Se and Te nanoparticles (e.g. Zhang et al. 2001; Prakash et al. 2009; Dhanjal and Cameotra 2010; Bajaj et al. 2012). The formation of selenium and tellurium nanoparticles can be associated with protein which affects the formation and growth of nanoparticles and can control their size and distribution (Dobias et al. 2011; Hunter 2014a,b). However, this is apparently the first time that selenium oxide and tellurium oxide have been found after metalloid oxyanion interaction with fungi, together with elemental selenium and tellurium. In addition, spindle-shaped tellurium particles in the nano-size range were observed in fungal culture supernatants, of length ~ 190 nm and width < 30 nm, most similar in size and shape. Rod-shaped tellurium nanoparticles have been observed with bacteria, inside cells or on cell surfaces (Oremland et al. 2004, Baesman et al. 2007, Pearce et al. 2011, Kim et al. 2012, Zare et al. 2012, Forootanfar et al. 2015, Borghese et al. 2016, Espinosa-Ortiz et al. 2017). *Bacillus selenitireducens* was capable of reducing tellurium as tellurate or tellurite to rosette-aggregated Te^0^ rods with particle sizes of 30 nm × 200 nm and selenium as selenite or selenate to Se^0^ with a spherical particle size of 200 nm (Oremland et al. 2004, Baesman et al. 2007). Previous research has also demonstrated that *Bacillus selenitireducens* (Baesman et al. 2007), *Bacillus* sp. (Zare et al., 2012), *Pseudomonas pseudoalcaligenes* (Forootanfar et al. 2015), *Rhodobacter capsulatus* (Borghese et al. 2016), *Shewanella oneidensis* (Kim et al. 2012), *Shewanella barnesii* (Baesman et al. 2007), *Bacillus beveridgei* (Pearce et al. 2011) and *Phanerochaete chrysosporium* (Espinosa-Ortiz et al. 2017) can generate Te nanorods, nanospheres and needle- and splinter-like nanoparticles.

Two important bioremediation or biorecovery approaches are involved in selenium and tellurium transformations by fungi: reduction and methylation. Microbial methylation of inorganic Se and Te oxyanions to volatile species offers an approach for bioremediation of metalloid-polluted soils which has been clearly demonstrated for Se (Zare et al. 2012; Nancharaiah and Lens 2015). Extracellular production of BioSeNPs and BioTeNPs are mainly carried out by Se- and Te-respiring bacteria through dissimilatory metalloid reduction. In contrast, intracellular production of BioSeNPs and BioTeNPs may comprise a detoxification mechanism for selenium and tellurium oxyanions (Nancharaiah and Lens 2015). However, elemental selenium and tellurium may show antimicrobial activity as these NPs have large specific surface areas with strong reactive sites, although elemental Se or Te are generally regarded as of low or no toxicity (Zare et al. 2012; Nancharaiah and Lens 2015). The size and shape of selenium and tellurium nanoparticles generated from fungi are dependent on various capping and dispersing agents (Dobias et al. 2011). Biosynthesis of extracellular nanoparticles is usually achieved in two steps, reduction and precipitation, followed by nucleation and aggregation of crystal structures: nanoparticles attached to fungal surfaces may perturb metabolic functions, such as respiration (Afkar et al. 2003). The reduction of selenium oxyanions in bacteria can be catalysed by reductases, including nitrite reductase, sulfite reductase and DMSO (dimethyl sulfoxide) reductase (Harrison et al. 1984; DeMoll-Decker and Macy 1993; Afkar et al. 2003). The SeO_4_^2−^-respiring bacteria *Thauera selenatis* and *Pseudomonas selenitipraecipitans* strain CA-5 were capable of reducing both SeO_3_^2−^ and SeO_4_^2−^ to Se^0^ through periplasmic NO_3_^−^ reductase activity (DeMoll-Decker and Macy 1993; Hunter and Manter 2009). However, in *Shewanella oneidensis* MR-1, which also has the ability to reduce SeO_3_^2−^ to Se^0^, SeO_3_^2−^-reducing ability had no direct connection to nitrate or nitrite reductase. Deletions of genes encoding nitrate reductase (*napA*), nitrite reductase (*nrfA*) and two periplasmic electron transfer mediators for anaerobic respiration (*mtrA and dmsE*) did not affect the ability to reduce selenium oxyanions (Li et al. 2014). The mechanisms of bioreduction of selenate and selenite to elemental selenium by bacteria has been extensively examined, but the biological processes responsible for selenite and tellurite reduction by fungi have been relatively neglected.

Particle sizes obtained from SEM measurements were lower than those estimated from SP-ICP-MS measurements. This is due to the fact that the particle sizes obtained by SP-ICP-MS are augmented substantially by the hydrated capping agents, such as proteins, and solvation effects. Selenium and tellurium nanoparticles generated by microorganisms have properties that are difficult to mimic by chemical and physical approaches (Espinosa-Ortiz et al. 2017). Fungal generated elemental selenium and tellurium tended to show a well-dispersed behaviour and the nanoparticles were not in direct contact even within aggregates, indicating stabilisation of the nanoparticles by a capping agent.

Oxidation of reduced selenium species may be relevant with respect to the availability of selenium as a trace element. Various works have indicated that microorganisms are capable of aerobic oxidation of Se^0^ and SeO_3_^2−^ in the soil. Microbial oxidation of elemental selenium occurred in soil slurries and bacterial cultures transformed elemental selenium into both selenite (SeO_3_^2−^) and selenate (SeO_4_^2−^), with selenite being the dominant product. This indicated that microbial oxidation in soils is partly constrained by adsorption of selenite on surfaces of soil components (Dowdle and Oremland 1998). Earlier evidence suggested that *Acidithiobacillus ferrooxidans* could use copper selenide oxidation as a source of energy (Torma and Habashi 1972). *Bacillus megaterium* can also oxidize Se^0^ to SeO_3_^2−^ with traces of SeO_4_^2−^ (Nancharaiah and Lens 2015). However, this is the first time that selected fungal strains have shown selenite oxide and tellurite oxide formation together with elemental selenium and tellurium. This implies that oxidation, reduction and methylation of selenium and tellurium species can occur during interaction with fungal species.

In conclusion, Se- or Te-species removal by fungi is accomplished through intracellular uptake or interaction with surface biomolecules such as extracellular proteins, amino acids and extracellular polymeric substances, while cellular biotransformation of these oxyanions leads to reduction to elemental selenium and tellurium or transformation to selenium oxide and tellurium oxide. The application of biologically induced, semi-synthetic production of selenium and tellurium nanoparticles, together with generation of selenium oxide and tellurium oxide could be a promising addition to current chemical or physical processes for nanoparticle production. Both elemental selenium and tellurium nanoparticles have been shown to protect organisms from DNA oxidation (Wang et al. 2007; Tran and Webster 2011; Huang et al. 2016), as well as possibly acting as promising antimicrobial and anticancer agents (Ahmad et al. 2015; Zonaro et al. 2015; Cremonini et al. 2016; Piacenza et al. 2017). Bioremediation of selenium- and tellurium-polluted environments and biorecovery of elemental SeNPs and TeNPs also suggest an environmentally sustainable choice to treat contaminated soils, groundwater, wastewater, leachates and sediments (Piacenza et al. 2017). A challenge in the biogenic production of selenium and tellurium nanoparticles is their purification from fungal biomass, because the formation of selenium and tellurium nanoparticles can be achieved both intracellularly and extracellularly: separation of particles from biomass without altering their properties, shapes and sizes is challenging. However, this work has demonstrated the secretion of Se or Te nanoparticles into the culture medium, and also their formation by reactions between selenite and tellurite and spent fungal culture supernatants. Further investigation could be focused on properties of biogenic selenium and tellurium NPs in terms of surface absorbing activity, reactivity, selectivity and sustainable treatment capability to optimise their industrial application potential for bioremediation and biorecovery.
